# The Pentose Phosphate Pathway in Yeasts–More Than a Poor Cousin of Glycolysis

**DOI:** 10.3390/biom11050725

**Published:** 2021-05-12

**Authors:** Laura-Katharina Bertels, Lucía Fernández Murillo, Jürgen J. Heinisch

**Affiliations:** Department of Genetics, Faculty of Biology/Chemistry, University of Osnabrück, Barbarastrasse 11, D-49076 Osnabrück, Germany; lbertels@uni-osnabrueck.de (L.-K.B.); lucfernandez@uni-osnabrueck.de (L.F.M.)

**Keywords:** carbohydrate metabolism, oxidative stress, reduction power, bioethanol, cancer

## Abstract

The pentose phosphate pathway (PPP) is a route that can work in parallel to glycolysis in glucose degradation in most living cells. It has a unidirectional oxidative part with glucose-6-phosphate dehydrogenase as a key enzyme generating NADPH, and a non-oxidative part involving the reversible transketolase and transaldolase reactions, which interchange PPP metabolites with glycolysis. While the oxidative branch is vital to cope with oxidative stress, the non-oxidative branch provides precursors for the synthesis of nucleic, fatty and aromatic amino acids. For glucose catabolism in the baker’s yeast *Saccharomyces cerevisiae*, where its components were first discovered and extensively studied, the PPP plays only a minor role. In contrast, PPP and glycolysis contribute almost equally to glucose degradation in other yeasts. We here summarize the data available for the PPP enzymes focusing on *S. cerevisiae* and *Kluyveromyces lactis*, and describe the phenotypes of gene deletions and the benefits of their overproduction and modification. Reference to other yeasts and to the importance of the PPP in their biotechnological and medical applications is briefly being included. We propose future studies on the PPP in *K. lactis* to be of special interest for basic science and as a host for the expression of human disease genes.

## 1. Introduction

For a long time investigations of central carbohydrate metabolism in yeast has raised comparatively little scientific interest. This has changed in the last few years with their increasing relevance for novel biotechnological processes and the enormous potential of the new discipline of synthetic biology [1,2]. Moreover, although the wine, beer, and baker’s yeast *Saccharomyces cerevisiae* holds a leading position not only in classical fermentations but also as a key model organism for eukaryotic cell biology [3,4], other “non-conventional” yeast species have been intensively studied as alternative microbial models and production organisms [5,6,7].

In this context, we decided to give an overview of the role of the pentose phosphate pathway, further abbreviated as PPP, and its constituting enzymes in yeast sugar metabolism, as compared to glycolysis. Besides the best studied yeast *S. cerevisiae*, we will primarily concentrate on the milk yeast, *Kluyveromyces lactis*, for its close relationship and model character [8], but also refer to *Candida albicans*, for its importance as an opportunistic human pathogen [9,10], and other yeast species, whenever data are available. For a broader perspective, including the importance for human physiology, interested readers are referred to excellent reviews on the PPP [11] and on yeast glycolysis [12,13]. Further reviews on the functions of the PPP in other fungi, plants, bacteria, the human immune system or the liver are also available [14,15,16,17,18,19].

## 2. Overview on the Reactions of the Pentose Phosphate Pathway (PPP)

**Figure 1:**
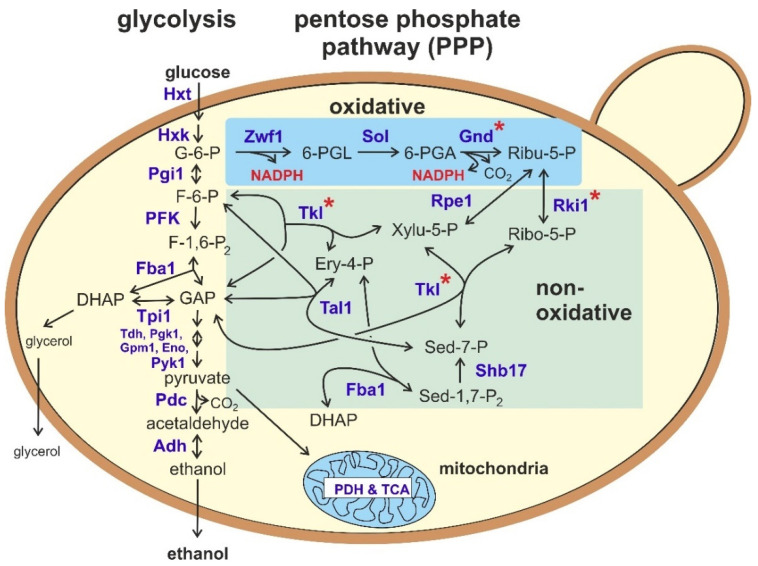
Overview of the reactions of the pentose phosphate pathway (PPP) and its connection to glycolysis and alcoholic fermentation. Enzymes are designated in bold blue letters, adopted from the nomenclature in *Saccharomyces cerevisiae*. Where more than one isozyme operates in this yeast, numbers behind the three-letter code have been omitted. Enzymes marked by a red asterisk are encoded by only one essential gene in the milk yeast *Kluyveromyces lactis*, i.e., deletions are not viable. One-headed arrows designate physiologically irreversible reactions, two-headed arrows reversible ones. The oxidative part of the PPP is shaded in blue, the non-oxidative part in green. Abbreviations of metabolites are: G-6-P = glucose-6-phosphate; F-6-P = fructose-6-phosphate; F-1,6-P_2_ = fructose-1,6-bisphosphate; GAP = glyceraldehyde-3-phosphate; DHAP = dihydroxyacetone phosphate; 6-PGL = 6-phosphogluconolactone; 6-PGA = 6-phosphogluconate; Ribu-5-P = ribulose-5-phosphate; Xylu-5-P = xylulose-5-phosphate; Ribo-5-P = ribose-5-phosphate; Ery-4-P = erythrose-4-phosphate; Sed-7-P = sedoheptulose-7-phosphate; Sed-1,7-P_2_ = sedoheptulose-1,7-bisphosphate. Enzyme/protein designations are: Hxt = hexose transporter; Hxk = hexokinase; Pgi1 = phosphoglucose isomerase; PFK = phosphofructokinase, written in capital letters, because it is a heterooctameric enzyme formed by four α- and four ß-subunits, encoded by the genes *PFK1* and *PFK2* [20]; Fba1 = fructose-1,6-bisphosphate aldolase; Tpi1 = triosephosphate isomerase; Tdh = glyceraldehyde-3-phosphate dehydrogenase (“triosephosphate dehydrogenase”); Pgk1 = phosphoglycerate kinase; Gpm1 = phosphoglycerate mutase; Eno = enolase; Pyk1 = pyruvate kinase; Pdc = pyruvate decarboxylase; Adh = alcohol dehydrogenase; Zwf1 = glucose-6-phosphate dehydrogenase (“Zwischenferment”); Sol = phosphogluconolactonase (“suppressor of *los1-1*”); Gnd = phosphogluconate dehydrogenase; Rki1 = ribosephosphate ketol isomerase; Rpe1 = ribulosephosphate epimerase; Tkl = transketolase; Tal = transaldolase; Shb17 = sedoheptulose-1,7-bisphosphatase; PDH = pyruvate dehydrogenase complex; TCA = tricarboxylic acid cycle.

The PPP is generally depicted with two branches (Figure 1): (i) the oxidative, essentially irreversible part, owing its designation to the generation of NADPH in two of its key reactions, and (ii) the non-oxidative, reversible part, in which metabolites are interconverted by the transaldolase and transketolase reactions. The latter also link the PPP to central carbohydrate metabolism by producing the glycolytic intermediates fructose-6-phosphate and glyceraldehyde-3-phosphate.

Both the PPP and the EMP (Embden–Meyerhof–Parnas pathway, or simply glycolysis) are ancient biological processes which ensure energy production from sugar sources and provide the building blocks for nucleic and amino acid syntheses. Thus, they operate and fulfill essential roles in cells from bacteria, fungi, plants and animals, including humans, where defects in enzymes of the PPP are associated with serious diseases (reviewed in [21,22]). In the context of pathway evolution, the oxidative part of the PPP appears to be more variable, as it is largely absent in archaea. There, a special ribulose monophosphate pathway (RMP) replaces the reactions of the oxidative PPP [23]. As an exception, both a glucose-6-phosphate dehydrogenase (G6PD) and a 6-phosphogluconate dehydrogenase (6PGD) have been identified in *Haloferax volcanii*, a member of the haloarchaea [24]. In addition to the classical reactions commonly depicted in textbooks, metabolome analyses in *S. cerevisiae* revealed that carbon can also be fed into the PPP independent of the oxidative branch. This is achieved via an activity of the glycolytic aldolase enzyme, Fba1, which condenses dihydroxyacetone phosphate with erythrose-4-phosphate to generate sedoheptulose-1,7-bisphosphate (Figure 1; [25]). The latter is then metabolized by a specialized, highly conserved bisphosphatase, Shb17.

In the following section, we will discuss the enzymes involved in the PPP one by one, comparing their features. Key parameters of the yeast enzymes and their encoding genes are also listed in Table 1.

### 2.1. The Oxidative Part of the PPP

#### 2.1.1. Glucose-6-phosphate Dehydrogenase

**Figure 2:**
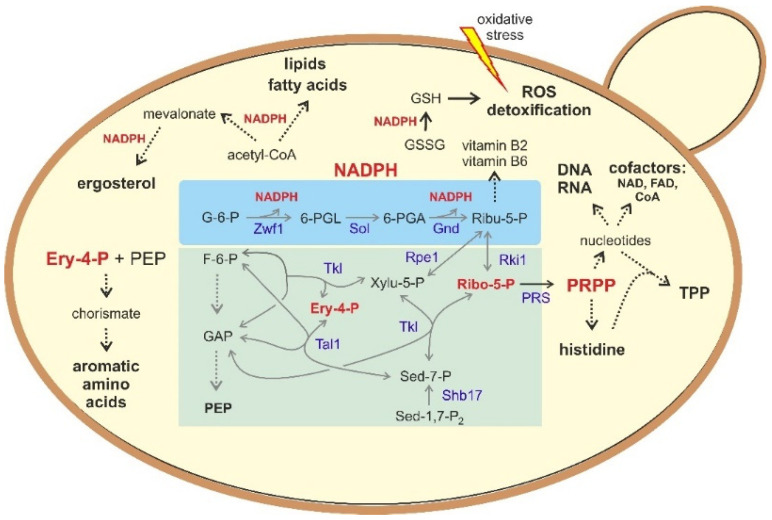
PPP metabolites and their role in biosynthetic processes and response to oxidative stress. Key metabolites of the PPP (central part shaded in blue and green) are highlighted in red and enlarged again in its periphery. Arrows with dotted lines indicate intermediate reactions not shown in detail. Abbreviations for the PPP are listed in the legend of Figure 1. Additional abbreviations are: PEP = phosphoenol pyruvate; PRS = phosphoribosylpyrophosphate synthase; PRPP = phosphoribosyl pyrophosphate; GSSG/GSH = oxidized and reduced forms of glutathione, respectively; ROS = reactive oxygen species; NAD = nicotinamide adenine dinucleotide; FAD = flavine adenine dinucleotide; CoA = coenzyme A; TPP = thiamine pyrophosphate; vitamin B2 = riboflavin; vitamin B6 = pyridoxal phosphate (see [47] for a review on vitamin synthesis in yeast).

Glucose-6-phosphate dehydrogenase (G6PD), discovered in the first half of last century by Otto Warburg as “Zwischenferment” (hence the common abbreviation Zwf; [39]), has certainly attracted the most interest of all the PPP enzymes, not least because its malfunction is related to an abundant human hereditary disease with an estimated 400 million cases worldwide, which most frequently results in hemolytic anemia (see [45,46], and references therein). As shown in Figure 2, catalysis by G6PD is the first reaction to generate NADPH and thereby provides a reduction power for the synthesis of fatty acids and lipids, as well as reducing glutathione required for the detoxification of reactive oxygen species (ROS).

In *S. cerevisiae*, the active enzyme appears to be a tetramer, whose dissociation into dimers and decrease in activity could be triggered by NADPH [48]. The subunits are encoded by the *ZWF1* gene, which has been cloned and deleted from the haploid yeast genome by various groups [26,28,29,30,49]. Deletions invariably show an increased sensitivity towards oxidative stress conditions; for example, cells are unable to grow in the presence of hydrogen peroxide. This can be largely attributed to the failure to produce sufficient NADPH for the reduction of glutathione needed to cope with increased ROS levels (Figure 2). Nevertheless, *ZWF1* gene expression appears to be fairly constitutive, i.e., it is not induced under oxidative stress [50]. Another phenotype of *zwf1* mutants is their dependence on an organic sulfur source reflected by their methionine auxotrophy, hence the synonymous designation of *ZWF1* as *MET19* [30]. Although not evident from the original work, this has also been attributed to the depletion of NADPH, as three molecules of this cofactor are required for the production of one methionine molecule [51,52]. Growth on rich medium under non-stress conditions remained unaffected by a *zwf1* deletion in several yeast strains [28,29,30], while being severely impaired in a strain more closely resembling those used for industrial purposes [26]. The chronological life span was also reduced in this genetic background, but not in others, as would be expected from the impaired production of reducing power and the concomitant failure to cope with internal ROS species produced by metabolism.

The *KlZWF1* gene has been cloned and deleted in the Pasteur-positive (Crabtree-negative) yeast *K. lactis*. The Pasteur effect is commonly interpreted as the downregulation of fermentation in the presence of oxygen, since respiration produces much higher ATP yields per molecule of glucose [53]. Ironically, this occurs in *K. lactis*, but not in *S. cerevisiae*, the yeast that Pasteur was actually working on [31]. There, high sugar concentrations lead to repression of respiration and a predominantly fermentative metabolism, independent from oxygen availability. This behavior is known as the Crabtree effect [54]. Despite these metabolic differences, *KlZWF1* gene expression appeared to be constitutive, as is observed in the *S. cerevisiae* homolog. The *Klzwf1* mutant showed a reduced biomass production on different carbon sources, suggesting that the enzyme activity is required for both fermentative and respiratory metabolism. KlZwf1 was found to occur both as a tetramer and as a dimer. Careful biochemical analyses indicated that conformational transitions, caused by the replacement of NADP^+^ for NADPH at an allosteric site in the subunits, trigger the formation of dimers of the tetrameric active enzyme and lead to an inhibition of its activity [32].

In *Candida albicans*, functional peroxisomal signal sequences were detected in CaZwf1 and also in the 6-phosphogluconate dehydrogenase CaGnd1 [55]. Thus, while a majority of these enzymes were cytosolic, approximately 10% and 5% of the proteins, respectively, were localized in peroxisomes, providing a means for detoxification of ROS produced during fatty acid degradation. In a recent work, heterozygous *CaZWF1/Cazwf1* mutants showed a reduced growth under hypoxic conditions and the authors state that the homozygous deletion would be lethal [56].

*ZWF1* genes have also been studied in *Kluyveromyces marxianus* [57], and the enzymes were purified from *Schizosaccharomyces pombe* [58] and *Candida utilis* [59]. Except for an unusual serine/threonine-rich region in the latter enzyme with unknown functional consequences, they seem to be similar to those from other yeasts.

#### 2.1.2. 6-Phosphoglucono Lactonase

G6PD produces 6-phosphoglucono lactone, which originally was believed to spontaneously convert to 6-phosphogluconate. However, the *S. cerevisiae* genome contains four genes for putative 6-phosphoglucono lactonases, named *SOL1-SOL4* for the ability of its founding member—*SOL1*—to partially suppress the phenotypes of a *los1-1* mutation when overexpressed. Only Sol3 and Sol4 show 6-phosphogluconlactonase activity and their overproduction does not suppress the *los1* mutant. Thus, Sol3 and Sol4 were proposed to function in the PPP, while Sol1 and Sol2 are involved in nuclear tRNA export [34]. Quadruple mutants are perfectly viable and only the *sol3* deletion has been found to be more sensitive to oxidative stress in a large-scale screen [34,60].

For *K. lactis*, only one gene representing a homolog of *SOL3* and *SOL4* exists in the genome sequence (using the yeast genome order browser [61], http://ygob.ucd.ie/, accessed on 25 March 2021), which may encode the 6-phosphoglucono lactonase, but has not yet been investigated. In analogy to *S. cerevisiae*, we assume that one other homolog of *SOL1* and *SOL2*, also found in the *K. lactis* genome, would encode a protein not related to carbohydrate metabolism.

#### 2.1.3. 6-Phosphogluconate Dehydrogenase

A second NADPH is produced in the oxidative PPP by the action of 6-phosphogluconate dehydrogenase (6PGD; Figure 1). Concomitantly, the C1 carbon from glucose is removed by decarboxylation, generating ribulose-5-phosphate, which feeds into the non-oxidative part of the pathway. In *S. cerevisiae*, due to the whole genome duplication, two encoding genes were found—*GND1* and *GND2*—with Gnd1 contributing approximately 80% of the enzyme activity [62]. Accordingly, while *gnd2* deletions do not display a strong phenotype under standard growth conditions, *gnd1* deletions grow more slowly on glucose media, and in some strain backgrounds, not at all [33]. Growth of the former strains is inhibited by hydrogen peroxide [28]. Neither the slow growth nor the sensitivity to oxidative stress are exacerbated by an additional deletion of *GND2*.

Only one homologous gene can be found in *K. lactis*, which has not undergone a whole genome duplication, and a *Klgnd1* deletion is lethal in the background of the sequenced type strain, i.e., spores from heterozygous diploids carrying the deletion do not produce colonies after tetrad analysis on rich medium with yeast extract, peptone, and glucose as a carbon source (this laboratory, unpublished results).

6PGD enzymes have been purified from *C. utilis* [63] and from *S. pombe*. The latter works as a tetramer in contrast to the dimeric enzymes from other yeasts [64].

### 2.2. The Non-Oxidative Part of the PPP

#### 2.2.1. Ribulosephosphate Epimerase

Null mutants in the sole gene for ribulosephosphate epimerase, *RPE1*, have been constructed in a haploid strain of *S. cerevisiae*. While rendering the cells devoid of any detectable D-ribulose-5-phosphate 3-epimerase activity, they displayed a reduced growth under standard conditions as well as on minimal media with glucose as a sole carbon source [33]. As expected, such strains are unable to grow on D-xylulose, which wild-type cells can utilize at a slow rate [65], since ribose-5-phosphate as the second pentose phosphate feeding into the PPP cannot be generated in the absence of the enzyme (Figure 1). As the mutants in the oxidative PPP, *rpe1* deletions proved to be hypersensitive towards hydrogen peroxide [28]. To our knowledge, data on mutants from other yeast species have not been published so far.

#### 2.2.2. Ribosephosphate Ketol Isomerase

The gene encoding ribosephosphate ketol isomerase, *RKI1*, is essential in *S. cerevisiae*, preventing the analysis of haploid null mutants [65]. The reason for this lethality remains elusive, given that the other PPP mutants, like *rpe1* reported above, are viable. A conditional mutant, *rki1*^R189K^, abolished more than 99% of the wild-type enzyme activity and rendered the cells auxotrophic for pyridoxine [38]. Biochemical analyses showed that this mutation affected the quaternary structure of the enzyme, so that the tetrameric form dissociated into dimers. In *K. lactis*, a *Klrki1* deletion, is also not viable (this laboratory, unpublished results). The gene appears to be essential in *C. albicans* as well, as only mutants with a heterozygous genotype, *CaRKI1/Carki1*, could be studied [56].

#### 2.2.3. Transketolase

*S. cerevisiae* carries two genes encoding putative transketolase isozymes: *TKL1* and *TKL2*. The lack of any detectable enzyme activity in vitro in crude extracts from a *tkl1* deletion clearly indicates that this gene encodes the major isoform [43]. However, the authors deduced that some activity of Tkl2 in vivo fulfills an important physiological function, as only *tkl1 tkl2* double mutants proved to be auxotrophic for aromatic amino acids, whose synthesis requires erythrose-4-phosphate as a precursor (Figure 2). Other phenotypes of the *tkl1* null mutant apparently depend on the genetic background of the strain employed, with various degrees of growth impairment on synthetic media [42,43,66,67]. A lack of growth on D-xylulose and an increased sensitivity against hydrogen peroxide is already displayed by the *tkl1* mutant, and is not enhanced by an additional *tkl2* deletion, further substantiating the view that *TKL1* encodes the major transketolase isoform [28,37,68].

The sole gene for transketolase in the *K. lactis* genome, *KlTKL1*, has been cloned and analyzed [27]. Interestingly, sequence analyses indicated that the transketolase genes from yeasts are more closely related to those from prokaryotes than from other eukaryotes. Expressed under the control of its original promoter, *KlTKL1* complemented the growth phenotypes of the *tkl1 tkl2* double deletion in *S. cerevisiae* and restored transketolase activity. However, attempts to construct a *Kltkl1* deletion were not successful at the time, provoking the assumption that the gene may be essential in this yeast. Unpublished data from our laboratory now confirm this hypothesis. Again, this is consistent with data from *C. albicans* indicating that the homozygous deletion in this diploid yeast is lethal [56].

#### 2.2.4. Transaldolase

Unlike the transketolase, a single *TAL1* gene encodes transaldolase in the *S. cerevisiae* genome. Accordingly, its deletion abolishes all enzymatic activity [41]. *NQM1*, a paralog present in the genome presumed to have originated from the whole genome duplication, seems to play a minor role and was studied mainly in a highly modified strain capable of xylose fermentation [69]. The *tal1* deletion is perfectly viable under standard growth conditions, indicating that all essential intermediates of the PPP can be formed in cells lacking this enzyme. However, accumulation of sedoheptulose-7-phosphate in the null mutant proves that the transaldolase reaction is required to maintain an equilibrium of metabolites in the non-oxidative PPP. The *TAL1* gene in wild-type cells is expressed constitutively [41,49]. As with the other PPP genes, *tal1* mutants are hypersensitive towards oxidative stress conditions [28,49]. The null mutant also requires supplementation with inositol, as observed in a large-scale screen [70]. A point mutation at lysine residue 144 also rendered the mutant catalytically inactive [37].

*K. lactis* also contains a sole gene encoding a transaldolase, *KlTAL1*, which has been cloned and deleted in a haploid strain background [44]. Like the transketolase gene, the wild-type gene from *K. lactis* was shown to restore enzyme activity in the respective *tal1* null mutant of *S. cerevisiae*, proving its functional homology. Combination of the *Kltal1* deletion with deletions in glycolytic genes was crucial in the assessment of the importance of the PPP for glucose utilization in *K. lactis*, as explained below in chapter 3. In *C. albicans*, the *CaTAL1* gene has been reported to be essential, but displays a certain degree of haploinsufficiency under conditions of limiting oxygen supply [56]. 

The existence of three transaldolase isoforms in *C. utilis* was suggested by early biochemical studies and was later deduced to originate from two encoding genes, whose products can form both homo- and heterooligomers [71].

#### 2.2.5. Sedoheptulose-1,7-bisphosphatase

For a long time, the reactions described so far were thought to cover the PPP in yeast and other organisms. However, metabolic flux analyses with modern mass-spectrometry analyses revealed another, hitherto unrecognized enzymatic activity in *S. cerevisiae*. Cells with a deletion of the previously uncharacterized open reading frame *YKR043c* showed a distinct accumulation of seven and eight carbon sugar phosphates, identified as sedoheptulose-1,7- and octulose-1,8-bisphosphate [25]. Apparently, sedoheptulose-1,7-bisphosphate (S1,7P_2_) is a metabolite that serves to replenish the PPP with carbon. Under growth conditions when there is little need for the reducing power generated by the oxidative part of the pathway, S1,7P_2_ is produced and then dephosphorylated by a specific bisphosphatase, Shb17. The enzyme seems to be widely conserved amongst the different biological kingdoms. In fact, homologs of the *SHB17* gene (renamed from *YKR043c*) of *S. cerevisiae* are present in the genomes of all other yeasts listed in the genome order browser (http://ygob.ucd.ie/ as of 25 March 2021 [61]), including *K. lactis*. This indicates that the encoded bisphosphatase serves an important biological function. Besides the accumulation of metabolites, no distinct growth phenotypes have been associated with the *shb17* deletion in *S. cerevisiae* so far. Interestingly, the authors proposed a new connection between glycolysis and the PPP, in that sedoheptulose-1,7-bisphosphate is formed by the aldolase, condensing dihydroxyacetone phosphate and erythrose-4-phosphate in a reversible reaction (Figure 1; [25]). Similarly, they propose that the octulose bisphosphates could be formed by condensation of DHAP with ribose-5-phosphate, an important reaction that also occurs in plants [72]. Sedoheptulose-7-phosphate has also been reported to be converted to the bisphosphate by a side reaction of pyrophosphate-dependent phosphofructokinases (PFK) from bacteria [73,74]. Evidence for such a generation of sedoheptulose-1,7-bisphosphate by the yeast ATP-dependent PFK has not been found.

## 3. Contribution of the PPP to Sugar Degradation

Early attempts to determine the relative contribution of the PPP to sugar consumption in *S. cerevisiae* relied on glucose molecules specifically labelled with ^14^C at different carbon atoms. They were based on determinations of the radioactive label in the carbon dioxide produced, which varies with the degradation pathway: while the CO_2_ liberated by the oxidative branch of the PPP carries the C1 carbon of the glucose molecule, purely glycolytic degradation and channeling into alcoholic fermentation through the pyruvate decarboxylase reaction generates CO_2_ only from C3 and C4 [75,76]. From such studies it was estimated that only approximately 2.5% of the glucose is metabolized through the oxidative PPP by *S. cerevisiae* under standard growth conditions [76]. This agrees well with genetic studies, which demonstrated that blocking any step in glycolysis renders the mutants incapable of growth on glucose as a sole carbon source [77].

By contrast, *K. lactis* cells lacking either the *KlPGI1* gene or the genes encoding the two subunits of the heterooctameric phosphofructokinase, *KlPFK1* and *KlPFK2*, grew well on glucose media, already indicating that the flux through glycolysis is not absolutely essential in this yeast [78,79]. In fact, *S. cerevisiae* cannot grow on glucose when it lacks Pgi1. This was attributed to glucose-6-phosphate being fed into the oxidative PPP, which would lead to the accumulation of NADPH that cannot be re-oxidized, because *S. cerevisiae* lacks transhydrogenase activities [80]. On the other hand, external mitochondrial transhydrogenases exist in *K. lactis*, so that an NADPH imbalance is not an issue [81,82]. Triple mutants of the type *Kltal1 Klpfk1 Klpfk2* could not use glucose as a sole carbon source, as did the single *Kltal1* or the double *Klpfk1 Klpfk2* deletions. It was thereby concluded that the PPP and glycolysis both have sufficient capacities for glucose metabolization in *K. lactis* [44]. Accordingly, other simultaneous blocks of glycolysis and PPP in *Klpgi1 Kltal1* and *Klpgi1 Klzwf1* double mutants likewise failed to grow on glucose as a sole carbon source [31,44]. Consistent with the notion of an important contribution of the PPP to glucose degradation in *K. lactis*, specific activities of key PPP enzymes, like KlTal1 and KlTkl1, were considerably higher than those of their counterparts in *S. cerevisiae* [27,44].

Reduced growth rates under hypoxic conditions of strains being heterozygous for various mutations in genes of the PPP in *C. albicans* would suggest that, in this opportunistic pathogen, the pathway also contributes significantly to glucose consumption [56].

It now has become possible to study metabolic fluxes directly by following the in vivo distribution of carbohydrates in growing yeast cells. These studies generally confirm that less than 2.5% of the glucose consumed by *S. cerevisiae* cells growing on synthetic medium (i.e., defined mineral medium with 2% glucose as a carbon source) are diverted into the oxidative part of the PPP. In rich medium containing yeast extract, peptone, and also glucose as a carbon source, less than 0.9% of the sugar is degraded by the PPP [83]. These percentages also vary with growth conditions and the state of the cell cycle the yeasts are in. Thus, entering the growth phase from a G_0_ state clearly requires the synthesis of nucleic acids for DNA replication and gene expression and triggers a transient increase in the carbon flux to be re-routed into the PPP [84]. In addition, a considerable portion of glycolytic metabolites can enter the non-oxidative PPP through the connections depicted in Figure 1. Taken these into account, 10–20% of the carbon atoms are redistributed to the PPP [25,33,85]. In contrast, and consistent with the genetic and biochemical data presented above, the contribution of the PPP to glucose degradation in *K. lactis* has been estimated to be much higher, reaching up to 40% [86].

Regarding its regulation, the PPP in *S. cerevisiae* has been proposed to participate in the fastest response to oxidative stress, reacting within the range of seconds [87]. This is owed to the links with glycolysis depicted in Figure 1. Thus, ROS rapidly inactivates the glycolytic enzymes glyceraldehyde-3-phosphate dehydrogenase (GAPDH, with isozymes encoded by three *TDH* genes) and triosephosphate isomerase (Tpi1), which is accompanied by the activation of NADPH production through Zwf1 [88]. This has been originally attributed to the accumulation of the precursor metabolites, especially of glucose-6-phosphate as a substrate. However, metabolome analyses suggested that it is rather the increased recycling of this sugar phosphate through the non-oxidative PPP caused by the limitation in glycolytic flux [89]. This hypothesis, although probably valid across the biological kingdoms [26], is based largely on data from human erythrocytes, rather than on studies in yeast [90]. In the blood cells, the rapid response to oxidative stress is enabled by the fact that the NADPH produced in the oxidative PPP is rapidly scavenged to produce reduced glutathione for detoxification of ROS [89]. Otherwise, the activity of G6PD would be effectively be inhibited to less than 1% of its capacity by NADPH and ATP [91]. Similar mechanisms are probably acting on the yeast enzymes, at least in *S. cerevisiae* and *K. lactis* [32]. Due to the reversibility of the reactions catalyzed by the enzymes of the non-oxidative PPP, a tight control of their activities is not expected. Consistent with the increased need for synthesis of nucleotides and aromatic amino acids (Figure 2), metabolite levels within the PPP were shown to be elevated during growth on synthetic as opposed to rich media [92].

At the level of gene expression, the zinc-dependent transcription factor Stb5 appears to be the major activator of genes encoding PPP enzymes [93,94,95]. These include *ZWF1*, *GND1*, *GND2*, and *TAL1*, but markedly not *RPE1*. Stb5 is also required for the proper induction of *SOL3* and *RKI1*. As expected, its binding to the promoters of many of these target genes is activated by the addition of diamide as an oxidative stress agent [95]. Although not induced by diamide, expression of *TKL1* also requires Stb5, as shown by its decreased transcript levels in a *stb5* deletion mutant. Since gene expression of non-PPP is hyper-activated in such deletions, the authors concluded that Stb5 may act either as a transcriptional activator or repressor, depending on the target gene. Notably, its binding reduces the expression of the glycolytic *PGI1* gene. The general function of Stb5 therefore lies in maintaining the NADPH balance [95].

## 4. Biotechnological Implications

### 4.1. Fermentation of Pentoses by S. cerevisiae

**Figure 3:**
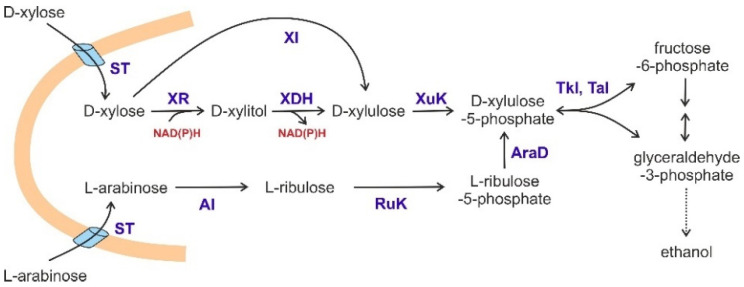
Heterologous pathways established to channel xylose and arabinose into the PPP and alcoholic fermentation. ST = sugar transporter, which can be either side activities of hexose transporters (e.g., Hxt7 or Gal2) existing in *S. cerevisiae*, or specific transporters for the respective pentose from heterologous sources [110,112]; XR = xylose reductase; XDH = xylitol dehydrogenase; XI = xylose isomerase; XuK = xylulokinase; AI = arabinose isomerase; RuK = ribulokinase; AraD = L-ribulose-5-phosphate-4-epimerase; Tkl = transketolase; Tal = transaldolase.

The wine, beer and baker’s yeast *S. cerevisiae* has been employed for thousands of years as a workhorse for the production of beverages from hexose sugars by mankind [96], to which first-generation bioethanol production has been added in the last decades [97,98]. Based on the long-term experience in these fermentation processes, and the ease of its genetic manipulation, *S. cerevisiae* has been applied to the conversion of alternative carbon sources for second-generation biofuels, especially from pentoses [99]. The latter are not only found abundantly in the waste-streams of the paper industry, but also form a major part of the biomass in plant-derived lignocellulosic material [100]. Two alternative approaches of heterologous gene expression have been used to convert xylose from such sources into xylulose-5-phosphate as a substrate that can be metabolized by the PPP, and, through its connection with glycolysis, ultimately be fermented to ethanol (Figure 3). (i) Early attempts relied on the heterologous expression of two genes from *Scheffersomyces stipitis* (then still called *Pichia stipitis*), which encode xylose reductase and xylitol dehydrogenase [101,102]. This resulted in the accumulation of xylitol, which was overcome by further manipulations of the cofactor requirements of the enzymes [103]. (ii) High-level expression of xylose isomerase genes from various sources avoided the imbalance of reduced cofactors, and, combined with overproduction of the native xylulokinase and elimination of an aldose reductase gene, the problem of xylitol production was resolved [104]. Xylose fermentation has been further improved by overproduction of endogenous Tkl1 in conjunction with heterologous Rki1 and Tal1 from *Kluyveromyces marxianus* [105]. In similar approaches, overexpression of *RPE1* was also shown to be beneficial [106,107,108]. Recent developments are aimed at the simultaneous fermentation of glucose and xylose, by additionally increasing the fermentation temperature in an extensively modified strain background [109]. In addition, arabinose was channeled into the PPP of *S. cerevisiae* by employing the Gal2 transporter and expression of three bacterial genes for its conversion into xylulyose-5-phosphate (Figure 3; [110], reviewed in [99]). Efficient fermentation of the two pentoses has also been considerably improved by the engineering of their transport into *S. cerevisiae*, as reviewed in [111].

Further details on the importance of pentoses and lignocellulosic sources for the production of next-generation biofuels, and the important role of the PPP for detoxification of oxidants like furfural produced in these processes, would go far beyond the scope of this overview, but have been extensively reviewed, e.g., in [99,100,109,111,113,114].

### 4.2. The PPP and Synthetic Biology in the Production of Value-Added Products

Besides bioethanol, alternative biofuels are being developed, which are based on the production of fatty acids, also serving as building blocks for oleochemicals for cosmetic purposes and as detergents and industrial lubricants [115,116]. As outlined in Figure 2, fatty acid synthesis requires reduction equivalents from the oxidative PPP. Thus, it is not surprising that overexpression of *STB5*, which encodes a positive transcription factor and enhances the expression of PPP genes, has been instrumentalized for this purpose [93].

Moreover, platform strains of *S. cerevisiae* are being developed, to increase the level of isoprenoids as precursors for the production of carotenoids, other nutraceuticals and food colorants in many industrial processes [117]. In fact, the production of the potent antimalarial drug artemisinin in yeast is based on the increased supply of these precursors [118]. Again, isoprenoid synthesis depends on an increased generation of NADPH from the oxidative PPP, so that overexpression of *ZWF1* has been combined with a reduced glycolytic phosphofructokinase activity to obtain a more reductive power [119].

Unlike the unwanted occurrence of xylitol as a by-product in bioethanol production from pentoses described above, xylitol is of value as a sweetener and a functional food additive, with proposed anticancerogenic effects. Heterologous expression of xylose reductase genes has been employed in this context for xylitol production in yeast [120].

The immense capacity of synthetic biology in relation to the PPP was demonstrated by the production of shinorine in yeast. Shinorine is a secondary metabolite naturally produced by cyanobacteria that protects against the damaging effects of UV, which can be used in sun creams as an environmentally friendly additive [121,122]. Since sedoheptulose-7-phosphate is a key precursor for shinorine synthesis, an *S. cerevisiae* strain already manipulated to efficiently grow on xylose by expression of the xylose assimilating genes from *S. stipitis* was employed [121]. In that work, biosynthetic genes for shinorine synthesis from *Nostoc punctiforme* were introduced into the platform strain, the native *TAL1* gene was deleted, and *TKL1* and *STB5* were overexpressed.

Manipulation of the PPP has also been employed to increase the supply of erythrose-4-phosphate for the generation of aromatic amino acids and the production of compounds derived from them, as exemplified in [123,124,125]. Other valuable products, such as butanol and butanediols, can be produced from erythritol as a platform chemical derived from erythrose-4-phosphate in the PPP, using waste glycerol as a growth substrate [126].

### 4.3. Non-Conventional Yeasts

One of the yeasts extensively studied for its ability to metabolize xylose, *S. stipitis* (*P. stipitis*), served as the first source for genes heterologously expressed in *S. cerevisiae* in bioethanol production [101,102]. In fact, expression of these genes and those of the PPP is strongly induced by growing *S. stipitis* on xylose as a sole carbon source [127]. Yet, due to the low yield and the general sensitivity of this yeast to ethanol, research has been mainly focused on adapting the knowledge gained to the more suitable *S. cerevisiae* [128]. 

The yeast *Pichia pastoris* (*Komagataella phaffii*) has long been employed for heterologous protein production due to its high capacity and its efficient secretion system [129]. However, vast overproduction of foreign proteins requires a high energy input and presents a metabolic burden, often resulting in reduced product yields. In this context, overexpression of genes encoding PPP enzymes, especially *ZWF1* and *SOL3*, were found to improve the production of human superoxide dismutase by almost a factor of four [130]. As similar problems with heterologous protein productions have been observed in other yeasts, including *S. cerevisiae*, the approach of manipulating the PPP seems promising.

Compared to its close relative *K. lactis*, *K. marxianus* has two important advantages for industrial applications: it can grow on xylose as a carbon source, although glucose and lactose are preferred, and it is more thermotolerant, i.e., it thrives at temperatures higher than 47 °C (see [131], and references therein). However, it should be noted that considerable genetic heterogeneity, including their ploidy, has been observed for different *K. marxianus* strains, thus complicating data analyses [132]. Nevertheless, extensive genetic manipulations and laboratory evolution have led to the development of strains efficiently fermenting xylose [133]. This so far has included an overexpression of either endogenous or heterologous *XYL1* and *XYL2* genes, encoding xylose reductase and xylitol dehydrogenase, as well as a reduction in the carbon flow to glycerol by deleting the *GPD1* gene. In an analogy to the results from bioethanol production from pentoses by *S. cerevisiae* described above, considerable progress by the concomitant overproduction of PPP enzymes may be beneficial in future approaches.

The oleaginous yeast *Yarrowia lipolytica* has attracted a lot of attention due to its ability to grow on and convert hydrocarbons and its remarkable resistance to toxic industrial by-products [13]. Moreover, it was found that the PPP is highly active in this yeast and with the rise of the CRISPR/Cas9 technology, it can now be readily manipulated (reviewed in [134]).

These are just a few examples demonstrating that non-conventional yeasts and their PPP are of great interest for ongoing and future biotechnological applications. For a larger overview on these and other yeast species in applied pentose metabolism, the reader is referred to [128].

## 5. Yeasts as Workhorses to Study PPP-Related Diseases

The PPP has attracted considerable attention for its role in human health. A summary of diseases related to malfunctions of its components is given in Table 2. 

Gene expression and/or activity of most of the PPP enzymes are upregulated in cancer cells, which involves different signaling pathways, with prominent components like PI3K/Akt, Ras, p53, and mTor (reviewed in [151]). The requirement for this increase in PPP capacity has been attributed to its role in providing precursors for fatty and nucleic acid synthesis needed for cell proliferation, DNA damage repair and survival, as well as the supply of NADPH to cope with oxidative stress [137]. The latter is also instrumental in patients with Diabetes mellitus type 2, for which both overexpression and deficiency of G6PD reduce the level of insulin secretion [136]. Moreover, a diminished response to oxidative stress participates in the development of neurodegenerative diseases like Parkinson’s disease (PD) [139], whereas amyloid ß and tau, two proteins associated with Alzheimer’s disease (AD), may be modified by glycosylation with PPP-derived D-ribose [138]. Accordingly, chemotherapies and other treatments can be impaired by alterations in the PPP. Its enzymes are thus promising drug targets for new therapeutic approaches (see [136,152], and references therein). Beyond that, an interesting observation links the NADPH production within the oxidative PPP to the regulation of the circadian clock in both mammalian cells and in flies. The disruption of the circadian clock in cancer cells was therefore also related to the PPP [153,154]. From a comparison with cyanobacteria, it was proposed that the circadian clock in mammalian cells acts as a regulating device between a proliferative and a homeostatic metabolism [153]. Readjusting the “timer” by targeting the activity of PPP enzymes could thus be a putative future approach in cancer therapy.

While there are many regulatory functions exerted by the PPP, the majority of investigations concentrated on the reaction catalyzed by G6PD, since it is the first and rate-limiting step providing reductive power [155]. Moreover, as briefly mentioned above, mutations in the encoding gene, located on the human X chromosome, constitute the most common cause of hereditary disease, which frequently results in severe hemolytic anemia [45,135]. On the other hand, the deficiency in human erythrocytes may also have a moderate protective effect against malaria infections [156]. The structure, allosteric regulation, and physiological role of G6PD has therefore been extensively studied. A central mechanism in this context is the feedback inhibition of the enzyme by NADPH, which keeps its capacity at about 1% maximum under non-stress conditions [91]. This is rapidly and dramatically increased upon application of oxidative stress, which triggers the scavenging of the reduced cofactor for detoxification purposes, thus relieving the enzyme inhibition [90]. Vice versa, activating glycolytic flux by increasing PFK activity leads to inhibition of G6PD affecting metastasis of melanoma cells [157].

While the structure of human G6PD was studied predominantly from enzymes purified from *E. coli*, it has also efficiently been produced in *S. cerevisiae* [158]. These investigations revealed that it is a dimer of dimers, in which the dissociation of the tetrameric form is triggered by NADPH binding to an allosteric site, which is different from the catalytic one. While studies on the quaternary structure of the enzyme from *S. cerevisiae* appeared to give somewhat conflicting results, KlZwf1 from *K. lactis* displayed striking similarities to its human homolog [32]. This offers the possibility of applying the results from the yeast to the human enzyme. More importantly, it implies that expression of the human gene and its mutant variants could ease their structural and biochemical characterization.

This is also true for the investigation of other PPP enzymes, which are much more rarely associated with hereditary diseases and have barely been studied in yeast systems. Thus, transketolase deficiencies were originally associated with their decreased affinity for binding the cofactor thiamine pyrophosphate (TPP). That this really causes the Wernicke-Korsakoff syndrome was questioned later [146]. More recent reports still suggest a relation of human transketolase to alcohol-induced, TPP-dependent symptoms [145], and it was found that mutations in the encoding gene can cause both developmental and congenital heart defects [144].

Finally, the biochemical properties of human transaldolase have been studied and compared to the enzymes from other sources [149]. In humans, deficiencies cause a rare autosomal disorder of carbohydrate metabolism, which has been confirmed in only 39 patients so far. This can result in liver malfunctions and cirrhosis, but also causes congenital heart disease [148,150]. 

Other rare reports on diseases related to PPP, including progressive leukoencephalopathy caused by RPI deficiency and organ defects triggered by liver malfunction due to mutations in a *TALDO* gene, are reviewed in [140]. This stresses the need for a simple and easy-to-handle eukaryotic expression system to advance the basic research in these cases, which we propose to be either *S. cerevisiae* or *K. lactis*. In this context, only an expression of human *G6PD* gene variants and the human *RPIA* gene in *S. cerevisiae* have been reported so far [143,158]. However, given the structural conservation, exemplified by the modeling of human transketolase based on the crystal structure of the yeast enzyme [159], heterologous expression of the other genes is not expected to pose any problem. In fact, G6PD genes from various organisms have been successfully expressed in *S. cerevisiae* [26], as were those encoding key glycolytic enzymes like human muscle phosphofructokinase from patients suffering from glycogen storage disease [160]. Although there may exist specific modifications or protein interactions of the enzymes in human cells that cannot be mirrored in yeasts, their eukaryotic nature is expected to come closer to the natural environment than bacterial expression systems. Moreover, such specific factors, if they exist, could even be identified using the power of yeast genetics.

## 6. Conclusions and Outlook

The pentose phosphate pathway has attracted revived attention not only in the model yeast *Saccharomyces cerevisiae*, but also throughout the biological kingdoms [11]. For yeast, this has been focused mainly on the fermentation of pentoses to bioethanol, increasingly through the use of synthetic biology. In this respect, we have probably only seen the tip of the iceberg, as more and more other value-added products will be produced based on the platform strains obtained in these studies, as shown by a few examples in paragraph 4.2.

With respect to basic research, and in the light of the importance of the PPP for carbohydrate metabolism in *K. lactis* as opposed to *S. cerevisiae*, it is surprising that very few studies have been dedicated to its genetics so far. As outlined above, only KlZwf1 and its mutants have been studied in some physiological detail [31,32], while beyond that only *Kltal1* null mutants were obtained [44]. This is probably due to the fact that genetic manipulations, while being basically similar to those of *S. cerevisiae*, are more time-consuming and laborious in *K. lactis* [8], and our preliminary results indicate that many of the genes encoding PPP enzymes may be essential in this yeast (Figure 1). Nevertheless, genetics of the PPP in *K. lactis* will be addressed in the near future and the findings are expected to be largely applicable to its close relative *K. marxianus*. Moreover, with respect to the carbon flow into and within the PPP, modern metabolomic approaches are expected to yield more similar results to human physiology in *K. lactis* than in *S. cerevisiae*, as the latter is too specialized in alcoholic fermentation. The higher similarity to mammalian cell physiology also recommends *K. lactis* as a heterologous host to produce and study human isoforms and variants. In contrast to most other non-conventional yeasts, *K. lactis* shares the advantage of a similar life cycle with *S. cerevisiae*, thus participating in the “power of yeast genetics” [8], while a congenic strain series can serve as a platform for molecular genetic manipulations [161]. We thus look forward to interesting developments in the near future and are positive that *K. lactis* will re-draw the attention of the scientific community as an alternative model organism to “the yeast” *S. cerevisiae*.

## Figures and Tables

**Table 1:** Features of PPP enzymes and their encoding genes in *S. cerevisiae* and *K. lactis*.

Enzyme	Yeast	Gene (Systematic Name)/Accession Number ^1^	Structure/ Identity ^2^	Cofactors	Specific Activity (mU/mg) ^3^	References
Glucose-6-phosphate dehydrogenase (G6PD) EC 1.1.1.49	*S. cerevisiae*	*ZWF1* (YNL241C) NC_001146.8	2(4) × 59 kDa	NADP^+^	100–180	[26,27,28,29,30]
	*K. lactis*	*KlZWF1* (KLLA0D19855g)	2(4) × 59 kDa 69%	NADP^+^	135–430	[26,27,31,32]
6-Phosphoglucono-lactonase (6PGL) EC 3.1.1.31	*S. cerevisiae*	*SOL4* (YGR248W) NC_001139.9 *SOL3* (YHR163W) NC_001140.6	(?) × 28 kDa (?) × 28 kDa	-	n.d.	[33,34]
	*K. lactis*	*KlSOL4* (KLLA0A05390g)	(?) × 28 kDa 45%/53%		n.d.	
6-phosphogluconate dehydrogenase (6PGD) EC 1.1.1.44	*S. cerevisiae*	*GND1* (YHR183W) NC_001140.6 *GND2* (YGR256W) NC_001139.9	2 × 52 kDa	NADP^+^	48^c^	[28,30,35,36]
	*K. lactis*	*KlGND1* (KLLA0A09339g)	(?) × 54 kDa 85%/81%	NADP^+^	n.d.	
Ribulosephosphate epimerase (RPE) EC 5.1.3.1	*S. cerevisiae*	*RPE1* (YJL121C) NC_001142.9	(?) × 26 kDa	-	1900–2200	[28,37]
	*K. lactis*	*KlRPE1* (KLLA0E15071g)	(?) × 26 kDa 69%			
Ribosephosphate ketol isomerase (RKI) EC 5.3.1.6	*S. cerevisiae*	*RKI1* (YOR095C) NC_001147.6	4 × 28 kDa	-	91	[37,38]
	*K. lactis*	*KlRKI1* (KLLA0C13541g)	(?) × 30 kDa 65%		n.d.	
Transketolase (TKL) EC 2.2.1.1	*S. cerevisiae*	*TKL1* (YPR074C) NC_001148.4 *TKL2* (YBR117C) NC_001134.8	2 × 74 kDa	TPP Mg^2+^	80–100	[27,39,40,41,42,43]
	*K. lactis*	*KlTKL1* (KLLA0B09152g)	(?) × 74 kDa 77%/70%		230–260	[27]
Transaldolase (TAL) EC 2.2.1.2	*S. cerevisiae*	*TAL1* (YLR354C) NM_001182243.1	2 × 38 kDa	-	45–73	[28,37,41,44]
	*K. lactis*	*KlTAL1* (KLLA0A02607g)	(?) × 36 kDa 75%		298	[44]
Sedoheptulose-1,7-bisphosphatase (SHB17) EC 3.1.3.37	*S. cerevisiae*	*SHB17* (YKR043C) NC_001143.9	2 × 31 kDa	Mg^2+^	n.d.	[25]
	*K. lactis*	*KlSHB17* (KLLA0E14961g)	(?) × 31 kDa 71%			

^1^ accession numbers are from GenBank. ^2^ identities of amino acid residues of the *K. lactis* homologs to their *S. cerevisiae* counterparts were obtained using alignments with ClustalW; if two isoforms exist in *S. cerevisiae*, identity values are given in the order of appearance. ^3^ specific enzyme activities are given in mU/mg protein; note that different methods of protein determination were employed in different works. n.d. = not determined; (?) the oligomer structure of the enzyme is unknown; molecular weights for Sol3, Sol4, and Shb17 from *S. cerevisiae,* and all of the *K. lactis* proteins were deduced from the translated gene sequences, except for KlZwf1, which was determined experimentally [32].

**Table 2:** Diseases related to malfunctions of PPP enzymes.

Enzyme	Disease	References
Glucose-6-phosphate Dehydrogenase (G6PD)	hemolytic anemia, diabetes, lung, liver, colorectal, prostate, and cervical cancer, leukemia, heart defects, Parkinson’s disease, Alzheimer’s disease	[45,135,136,137,138,139,140]
6-Phosphoglucono-Lactonase (6PGL)	metastases in bones originating from breast cancer	[141]
6-Phosphogluconate Dehydrogenase (6PGD)	lung and brain cancer	[136,137]
Ribosephosphate Isomerase (RPI)	pancreatic cancer and leukoencephalopathy	[136,142,143]
Ribulosephosphate Epimerase (RPE)	pancreatic cancer	[136]
Transketolase (TKL)	lung, liver and pancreatic cancer, Wernicke-Korsakoff syndrome, heart defects	[136,144,145,146]
Transaldolase (TAL)	lung cancer, liver cirrhosis, anemia, thrombocytopenia, heart defects, renal malfunction and neonatal edema multiple sclerosis, rheumatoid arthritis	[136,140,147,148,149,150]

## Data Availability

Not applicable.
